# H3Africa multi-centre study of the prevalence and environmental and genetic determinants of type 2 diabetes in sub-Saharan Africa: study protocol

**DOI:** 10.1017/gheg.2015.6

**Published:** 2016-03-08

**Authors:** K. Ekoru, E. H. Young, C. Adebamowo, N. Balde, B. J. Hennig, P. Kaleebu, S. Kapiga, N. S. Levitt, M. Mayige, J. C. Mbanya, M. I. McCarthy, O. Nyan, M. Nyirenda, J. Oli, K. Ramaiya, L. Smeeth, E. Sobngwi, C. N. Rotimi, M. S. Sandhu, A. A. Motala

**Affiliations:** 1 Department of Medicine, University of Cambridge, Cambridge, UK; 2 Genetic Epidemiology Group, Wellcome Trust Sanger Institute, Hinxton, Cambridge, UK; 3 Institute of Human Virology, Abuja, Nigeria; 4 Department of Epidemiology and Public Health, Institute of Human Virology and Greenebaum Cancer Center, University of Maryland Baltimore School of Medicine, MD, USA; 5 CHU Donka, University of Conakry, Non Communicable Disease Unit, Ministry of Health, Conackry, Guinea; 6 MRC International Nutrition Group at MRC Keneba, MRC Unit, The Gambia; 7 MRC International Nutrition Group, London School of Hygiene and Tropical Medicine, UK; 8 MRC/UVRI Uganda Research Unit on AIDS, Entebbe, Uganda; 9 Mwanza Intervention Trials Unit/NIMR, Mwanza, Tanzania; 10 University of Nigeria Teaching Hospital, Enugu, Nigeria; 11 Division of Diabetic Medicine and Endocrinology, Department of Medicine, University of Cape Town, Cape Town, Chronic Diseases Initiative in Africa, South Africa; 12 National Institute for Medical Research, Dar es Salaam, Tanzania; 13 Faculty of Medicine and Biomedical Sciences, University of Yaounde 1, Yaounde, Cameroon; 14 Oxford Centre for Diabetes, Endocrinology and Metabolism, University of Oxford, Churchill Hospital, Old Road, Headington, Oxford, UK; 15 Wellcome Trust Centre for Human Genetics, University of Oxford, Roosevelt Drive, Oxford, UK; 16 Oxford NIHR Biomedical Research Centre, Churchill Hospital, Old Road, Headington, Oxford, UK; 17 Edward Francis Small Teaching Hospital, School of Medicine, University of The Gambia, Banjul, The Gambia; 18 Malawi Epidemiology and Intervention Research Unit, Lilongwe, Malawi; 19 Department of Medicine, Muhimbili University of Health and Allied Sciences, Dar es Salaam, Tanzania; 20 Faculty of Epidemiology and Population Health, London School of Hygiene and Tropical Medicine, London, UK; 21 Center for Research on Genomics and Global Health, National Human Genome Research Institute, NIH, Bethesda, MD, USA; 22 Department of Diabetes and Endocrinology, Nelson R. Mandela School of Medicine, University of KwaZulu-Natal, Durban, South Africa

**Keywords:** Epidemiology, genetics, H3Africa, sub-Saharan Africa, type 2 diabetes

## Abstract

The burden and aetiology of type 2 diabetes (T2D) and its microvascular complications may be influenced by varying behavioural and lifestyle environments as well as by genetic susceptibility. These aspects of the epidemiology of T2D have not been reliably clarified in sub-Saharan Africa (SSA), highlighting the need for context-specific epidemiological studies with the statistical resolution to inform potential preventative and therapeutic strategies. Therefore, as part of the Human Heredity and Health in Africa (H3Africa) initiative, we designed a multi-site study comprising case collections and population-based surveys at 11 sites in eight countries across SSA. The goal is to recruit up to 6000 T2D participants and 6000 control participants. We will collect questionnaire data, biophysical measurements and biological samples for chronic disease traits, risk factors and genetic data on all study participants. Through integrating epidemiological and genomic techniques, the study provides a framework for assessing the burden, spectrum and environmental and genetic risk factors for T2D and its complications across SSA. With established mechanisms for fieldwork, data and sample collection and management, data-sharing and consent for re-approaching participants, the study will be a resource for future research studies, including longitudinal studies, prospective case ascertainment of incident disease and interventional studies.

## Background

Type 2 diabetes (T2D) is a major cause of premature mortality and morbidity worldwide. In sub-Saharan Africa (SSA), T2D is an emerging epidemic with a current prevalence of around 1–6% [1]. In this region, it is estimated that 22 million adults were living with diabetes in 2013; this figure is expected to be more than double by 2035, representing the largest proportional increase in the burden of diabetes of any region in the world [2, 3]. The disorder presents a major health problem in SSA, competing for limited health resources with infectious diseases and other emerging non-communicable diseases (NCDs) such as cardiovascular disease [1, 4–7].

Although, several established and emerging risk factors for T2D and cardiovascular diseases in Africa are similar to those found in other parts of the world, the magnitude and distribution of these factors may differ, and have not been fully studied in SSA in a large-scale epidemiological context [4]. Population growth and the concomitant rise in life expectancy only partly explain the increase in diabetes and other chronic diseases. Many of the known risk factors for NCDs in high-income countries are the same for low- and middle-income countries, including smoking, alcohol and obesity; however, there is an additional impact of rapid urbanisation and globalisation and the associated trends towards unhealthy lifestyles. Additionally, the role of endemic infections on the aetiology of T2D, as well as on the prevalence and spectrum of diabetic complications has not been clarified in SSA [6].

In addition to these environmental and lifestyle risk factors, genetic predisposition towards T2D may provide additional insights into the differences in T2D prevalence observed between populations in SSA. At present, there are around 100 loci for which there is robust (genome-wide significant) evidence of association with traits related to T2D, including obesity and fasting hyperglycaemia, identified in predominantly European and Asian populations. However, the relevance of many recent genomic findings to populations in SSA has not been systematically studied. Given the marked genomic diversity among populations in SSA, understanding the genomic basis of T2D, its complications, and its risk factors in populations of African descent is likely to provide additional insights into disease aetiology and potential therapeutic strategies [8, 9]. These observations highlight the need for epidemiological studies with the statistical resolution to reliably assess the burden and epidemiology of T2D and inform potential preventative and therapeutic strategies relevant to SSA.

**Fig. 1.**
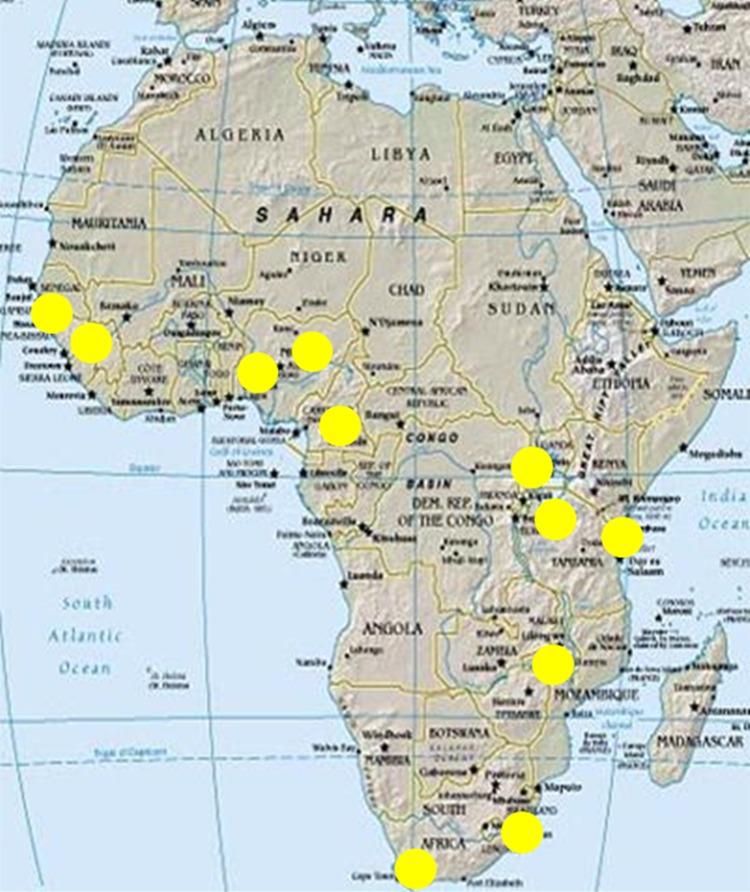
Geographical distribution of study sites in the H3Africa type 2 diabetes study.

Therefore, we have designed a multi-country study comprising 11 study sites located across eight countries in SSA to investigate the epidemiology and aetiology of T2D (Fig. 1 and Table 1). Each participating study site will collect cases of participants with T2D drawn from hospital clinics and healthcare facilities, and conduct a cross-sectional population-based survey to recruit controls. In total, across all study sites, we aim to recruit approximately 6000 cases of T2D and 6000 controls. Using harmonised and standardised tools for recruitment and data collection, we will be able to estimate the prevalence of T2D across SSA and conduct region-wide pooled analyses using comparable data, in order to identify risk factors and their relative contribution to T2D. This study will thus create a large-scale epidemiological and genomic research framework for improving our understanding of the burden and risk factors for diabetes, which may inform health service planning and prevention strategies across Africa. The study will also provide insights into the molecular and pathophysiological basis of diabetes in African populations and hence contribute to medical genomics efforts globally.

**Table 1.** Coordinating institutions and research partners for the H3Africa T2D study. The study comprises 11 sites in SSA, where field work is undertaken, together with research partners from the UK and the USA

Country	Coordinating institution
Field study sites	
Cameroon	Health of Populations in Transition, University of Yaoundé 1
The Gambia	MRC Keneba, MRC Unit, The Gambia and University of The Gambia
Guinea	CHU Donka, University of Conakry, Non Communicable Disease Unit, Ministry of Health
Malawi	Malawi-Liverpool-Wellcome Trust Unit, Blantyre
Nigeria	University of Nigeria Teaching Hospital, Enugu
Nigeria	Institute of Human Virology, Abuja
South Africa	Faculty of Health Sciences, University of Cape Town
South Africa	Nelson R Mandela School of Medicine, University of KwaZulu-Natal, Durban^a^
Tanzania	National Institute for Medical Research, Dares Salaam, Hindu Mandal Hospital, Muhimbili University of Health and Allied Sciences, Muhimbili National Hospital, and Nelson Mandela Institute
Tanzania	Mwanza Intervention Trials Unit/NIMR Tanzania
Uganda	MRC/UVRI Uganda Research Unit on AIDS, Entebbe
Research partners	
UK	Cambridge University; Wellcome Trust Sanger Institute; MRC Epidemiology Unit, Cambridge
UK	London School of Hygiene and Tropical Medicine
UK	Wellcome Trust Centre for Human Genetics; MRC Centre for Global Health Genomics, Oxford University
USA	National Human Genome Centre, Howard University, Washington; National Human Genome Research Institute, National Institutes of Health; Case Western Reserve University

a The principal investigator for the study – Prof Ayesha Motala –  is based at this study site.

## Study aims and objectives

The overarching aim of the study is to assess the burden and aetiological characteristics of T2D in adults in SSA, using large-scale population-based approaches. Within this aim, we have two study objectives:

1. To assess the burden, spectrum and environmental and genetic determinants of T2D among adults in SSA. This aim will be addressed across all 11 study sites.
2. To characterise the prevalence, distribution and environmental and genetic determinants of microvascular complications of diabetes among adults with T2D in SSA. To address this aim, nephropathy will be assessed across all 11 study sites. Diabetic retinopathy will be assessed in a sub-set of four study sites (Blantyre, Malawi; Abuja, Nigeria; Cape Town, South Africa; and Durban, South Africa).

## Methods

### Study sampling frame

**Fig. 2.**
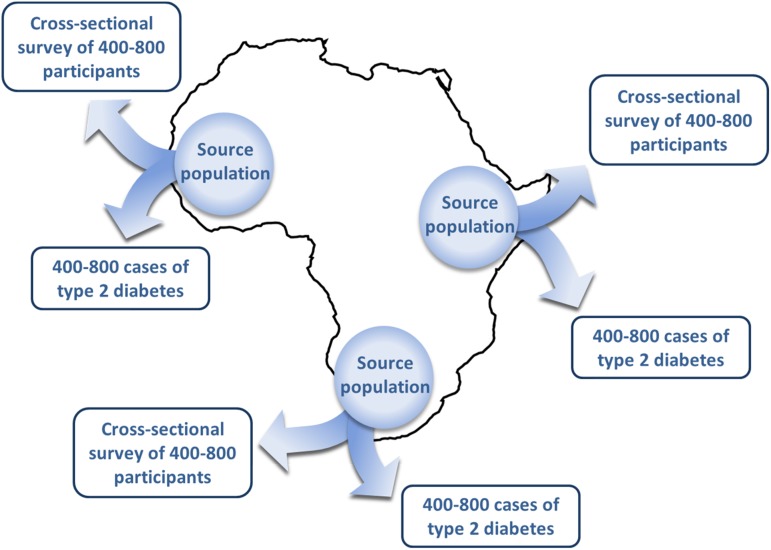
Schematic of multi-country case collection and cross-sectional survey design.

**Fig. 3.**
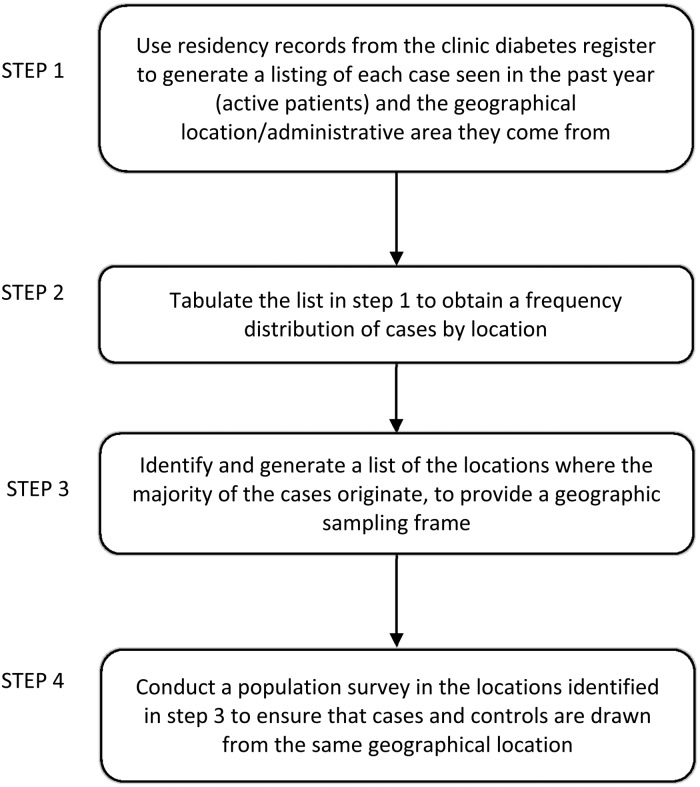
Generating a geographical sampling frame at each site.

Each study site will generate a geographical sampling frame for the case collection and population-based survey. First, using residency records from diabetes registers in hospital clinics and healthcare facilities, each site will generate a list of the diabetes patients seen in the preceding 12 months and the geographical location or administrative region they come from. Using this information, a list will be created of the specific locations from where the majority of patients originate, to provide a geographical sampling frame for recruitment of cases and controls into the study (Figs 2 and 3).

### Definitions

Clinically diagnosed T2D cases, based on data from patient medical records, will be defined according to current American Diabetes Association and World Health Organization (WHO) definitions [10, 11]: fasting plasma glucose (FPG) ≥7.0 mm/ (≥126 mg/dl) or 2-h post-load plasma glucose (2 h-PG) ≥11.1 mm/l (≥200 mg/dl) during oral glucose tolerance test (OGTT) or symptoms of diabetes and random plasma glucose ≥11.1 mm/l (≥200 mg/dl) or on treatment for diabetes (based on medical records or self-report). HbA1c will not be used to define diabetes in this study because its use has not been validated in this population, although it will be measured for comparison with glucose-based criteria. Presence or absence of diabetic retinopathy will be assessed according to the Liverpool Diabetic Eye Study grading for both retinopathy and macular exudates [12]. Microalbuminuria will be defined as albumin-to-creatinine ratio >2.0 mg/mm [13]. These case definitions will be used across all study sites to ensure consistency and comparability of data.

### Recruitment of T2D cases

In each study site, cases of T2D who live in the study geographical sampling frame will be recruited from hospital clinics and healthcare facilities using predefined inclusion and exclusion criteria (Table 2). Patients with T2D will be identified from patient registers and their clinical details entered into a case review dataset, to be assessed for eligibility before being invited to participate in the study. Eligibility will be independently monitored by senior clinicians from two of the collaborating institutes, who will receive and review the case review datasets from each study site to ensure that inclusion and exclusion criteria – in particular, diagnostic criteria for T2D – are applied consistently across all study sites. Individuals who agree to participate in the study will be required to provide written informed consent before recruitment (online Supplementary Appendix 1a); at study sites participating in the retinopathy sub-study, individuals will additionally consent to undergo digital fundus photography to assess and characterise the presence and severity of diabetic eye disease (online Supplementary Appendix 1b).

**Table 2.** Inclusion and exclusion criteria for cases of T2D and population-based controls

	Inclusion criteria	Exclusion criteria
Case of T2D	Age ≥25 years Clinically diagnosed T2D^a^ Individual of African origin (Black) Signed informed consent	Pregnant women^b^ Diabetes classified other than T2D or doubt as to classification Resident outside the geographical sampling frame for the relevant study site Self-defined ethnic group regarded as other than African (Black) Unable to give informed consent
Population-based controls	Age ≥18 years Individual of African origin (Black) Signed informed consent	Pregnant women^b^ Resident outside the geographical sampling frame for the relevant study site Self-defined ethnic group regarded as other than African (Black) Unable to give informed consent

T2D, type 2 diabetes.

a T2D defined according to current World Health Organization and American Diabetes Association guidelines – see methods section.

b Women can participate in the study from six months after childbirth.  The minimum age for participation in the population survey has been set at 18 to obtain a broadly representative sample of the underlying adult population. This will allow the opportunity to align controls to other case series that could potentially be collected in the context of future studies of other diseases and traits.

### Recruitment of population-based controls

In parallel to case recruitment, each study site will conduct a population-based cross-sectional survey to recruit controls within the geographical sampling frame to ensure the cases and controls are recruited from the same geographical region. Using standardised procedures across all sites, the surveys will adopt a multi-stage sampling design: the primary sampling units (clusters) are administrative units that are selected with probability proportional to size, and households are the secondary units selected by simple random sampling. Next, based on predefined inclusion and exclusion criteria (Table 2), eligible adults from the selected households will be invited to participate in the study. For the population-based surveys we will opt to use minimal inclusion/exclusion criteria in order to obtain a broadly representative sample of the population. This approach will allow the opportunity to align controls to other cases that could potentially be collected in the context of future proposals focusing on other diseases and traits.

Individuals, who wish to take part will be invited to attend static or mobile clinical hubs after an overnight fast of at least 8 h, and will be asked to provide written informed consent (online Supplementary Appendix 1c). As described later, the diabetes status of all survey participants (regardless of any self-reported medical history of T2D) will be established by undergoing a 75 g OGTT. Based on these OGTT results, participants without T2D will be used as controls for the study, while those found to have T2D will be added to the cases at analysis.

### Sample size and statistical power

A total of just over 12 000 participants will be recruited across all sites (with a minimum of 400–800 cases and an equal number of controls per site). In practice, at each study site the number of survey participants will be oversampled by around 10%, in order to obtain a number of controls equal to the number of cases based on an estimated prevalence of T2D of 10% among adults in the survey. Thus, the total survey sample size across all sites will be about 7000 participants. The sample size for the diabetic retinopathy sub-study will be approximately 2000 individuals. Other complications, namely microalbuminuria will be assessed among T2D cases across all sites, and will have a sample size of 6000.

**Fig. 4.**
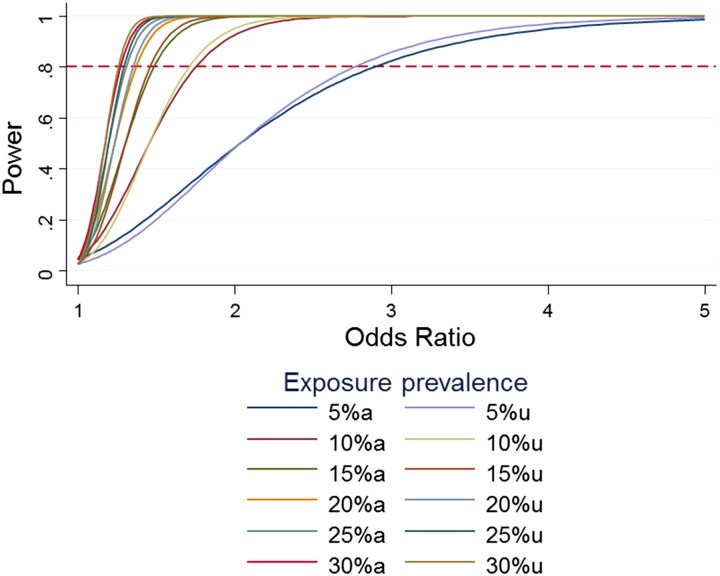
Power for a range of odds ratios and prevalence of exposure, with and without adjustment for clustering.

With a survey sample size of 7000 we will estimate the prevalence of T2D with high precision. For example, given a true prevalence of 10% and design effect (deff) of 3 or 4, we will estimate prevalence in the survey to within ±1.2% or ±1.4%, respectively. The precision will be greater with lower deffs: precision will be within ±0.7, ±0.9 and ±1.0% for deffs equal to 1, 1.5 and 2, respectively. Risk factors for T2D will be assessed using logistic regression based on 6000 cases and 6000 controls. The study will have more than 80% power of detecting odds ratios (ORs) as low as 1.5. Any loss of statistical power due to clustering of individuals within households is insignificant, as demonstrated by the near coincidence of power-effect size graphs with and without adjustment for clustering (Fig. 4).

### Data collection

Using the standardised procedures across all sites, we will collect detailed information from all study participants (T2D cases and survey participants) on health, lifestyle, medical and socioeconomic indices, as well as biophysical measurements, biosample collections for markers of non-communicable and infectious diseases, and genetic information. To maximise efficiency and convenience for participants, all data and samples are collected in a single study visit. Details of data and sample collection are shown in Tables 3 and 4. Questionnaire data will be collected using an electronic data capture system [electronic questionnaire (EQ)] developed by the African Partnership for Chronic Disease Research (APCDR) (http://www.apcdr.org/). To increase accuracy and efficiency of data collection, the EQ has built-in data checks, including double entry of numbers, plausibility of answers and appropriate numerical limitations, which have been described in detail elsewhere [14]. The EQ is administered through face-to-face interview and is based on the standardised and validated WHO STEPwise approach to Surveillance (STEPS) tool designed for the collection of chronic disease risk factors [15]. The questionnaire is available on request.

**Table 3.** Data collection components in the H3A T2D study

All participants	
Demographic	Age Sex Marital status Ethno-linguistic group Migration history
Participant medical history	Hypertension, dyslipidaemia, coronary heart disease Stroke, renal disease, diabetes-related eye disease HIV status, TB, malaria and other chronic infections (such as HCV), ART use
Lifestyle	Diet, physical activity, exercise Smoking, alcohol consumption
Socioeconomic	Level of education Years spent in school Household size Household income Occupation
Biophysical measurements	Height, weight Waist circumference, hip circumference Blood pressure
First degree family history	Diabetes, hypertension, dyslipidaemia, coronary heart disease
Survey participants only:	
Participant medical history	T2D
T2D cases and survey participants with self-reported T2D:	
Participant medical history^a^	Date of diagnosis of T2D Age at diagnosis of T2D Current therapy for diabetes Date of commencement of insulin therapy (if applicable) Self-reported presence of known microvascular complications

ART, antiretroviral therapy; HCV, hepatitis C virus infection; T2D, type 2 diabetes; TB, tuberculosis..

a For cases, these data are obtained from medical records captured in the case review form.

**Table 4.** Schedule of biosample collection. The list indicates the planned use for a single microvial derived from each sample collected. Remaining microvials are banked, both at the local laboratories and at the central biorepository, for long-term storage and future testing

	Sample collected	Use of samples
Collected at baseline, 0 min, from all participants (fasting)	10 ml plain serum blood tube	Biochemistry, serology & insulin and C-peptide
	5 ml or 6 ml EDTA whole blood tube^a^	DNA extraction
	2 ml EDTA whole blood tube	Full blood count
	2 ml EDTA whole blood tube	HbA1c
	10 ml EDTA whole blood tube	Plasma extraction for viral genomics
	2 ml NaF blood tube	Fasting plasma glucose
	Spot urine collection	Albumin-to-creatinine ratio, urinary Na and K
Collected at 120 min (non-fasting) from survey participants only	4 ml plain serum blood tube	Insulin and C-peptide
	2 ml NaF blood tube	Plasma glucose

a Volume of whole blood EDTA tube for DNA extraction will depend on local availability.

A range of biophysical measurements are taken (Table 3) with calibrated and validated equipment, using standardised procedures to ensure consistency and harmonisation of data across study sites. These data are also collected as part of the EQ dataset. Additionally, for T2D cases in the diabetic retinopathy sub-study, digital fundus photography will be used to assess the presence and severity of diabetic retinopathy and maculopathy. Photographic images will be graded at a central retinopathy hub in Durban, South Africa, using the Liverpool Diabetic Eye Study protocol. Venous blood and untimed spot urine samples will be collected from all T2D cases and survey participants after a preceding overnight fast of 8–12 h (Table 4). Further, all survey participants (including those with self-reported diabetes) will have a fasting finger prick blood glucose test and also undergo an OGTT (regardless of finger prick glucose result), requiring additional blood samples to be taken 2 h after the 75 g oral glucose load. Blood and urine samples will be stored in cold boxes maintained at 4–8 °C using standardised procedures, until transported to a laboratory on the same day as sample collection.

### Sample processing, biobanking and management

At each study site, blood and urine samples will be transported from the field to the local laboratory for further processing and analysis or storage, full details of which are available on request. In brief, full blood count is measured at the local laboratories, on the day of sample collection. The ethylenediaminetetraacetic acid (EDTA) whole blood samples (5 or 6 ml) for genomic analyses will be transferred to cryotubes for storage at −80 °C, before being shipped to the central biorepository in Uganda for the subsequent DNA extraction and next-generation genotyping and sequencing. Using standardised procedures, all other samples will be processed at the local laboratories for storage, including centrifugation, separation into serum and plasma and aliquoting into linked-anonymised barcoded microvials. Derived from each original collection tube, a set of microvials is sent to the central biorepository, whilst ensuring that back-up microvials are banked at the local laboratory indefinitely.

Apart from full blood count, all other laboratory tests and analyses will be conducted at the central biorepository which is located at the Medical Research Council/Uganda Virus Research Institute in Entebbe, Uganda. Samples are shipped to the central biorepository on dry ice, and are stored at −80 °C immediately on arrival. When sufficient numbers of samples have been received from different sites, batches of samples are selected, thawed and tested following standardised procedures (Table 4). For each assay run, samples from a number of different sites will be selected for testing in order to reduce bias in the biomarker measurements. Therefore, biochemical assays will be done randomly with respect to study site and case/control status. Samples for each participant will also be banked at the central biorepository as a resource for future research. Access to banked samples will follow standardised procedures and will be governed by the Biospecimen and Data Access policies of Human Heredity and Health in Africa (H3Africa) consortium (h3africa.org).

### DNA extraction and generation of genomic data

From each study participant, the central biorepository hub will receive an EDTA whole blood cryotube for DNA extraction. The DNA will be extracted and divided into three samples: one for genotyping and sequencing, a second sample to be returned to the local site, and a third sample for biobanking at the central biorespository hub. Generation of genomic data – including next-generation sequencing, genotyping arrays and imputation – will be undertaken in several established and internationally competitive genome centres, such as the Wellcome Trust Sanger Institute, UK.

### Data management

**Fig. 5.**
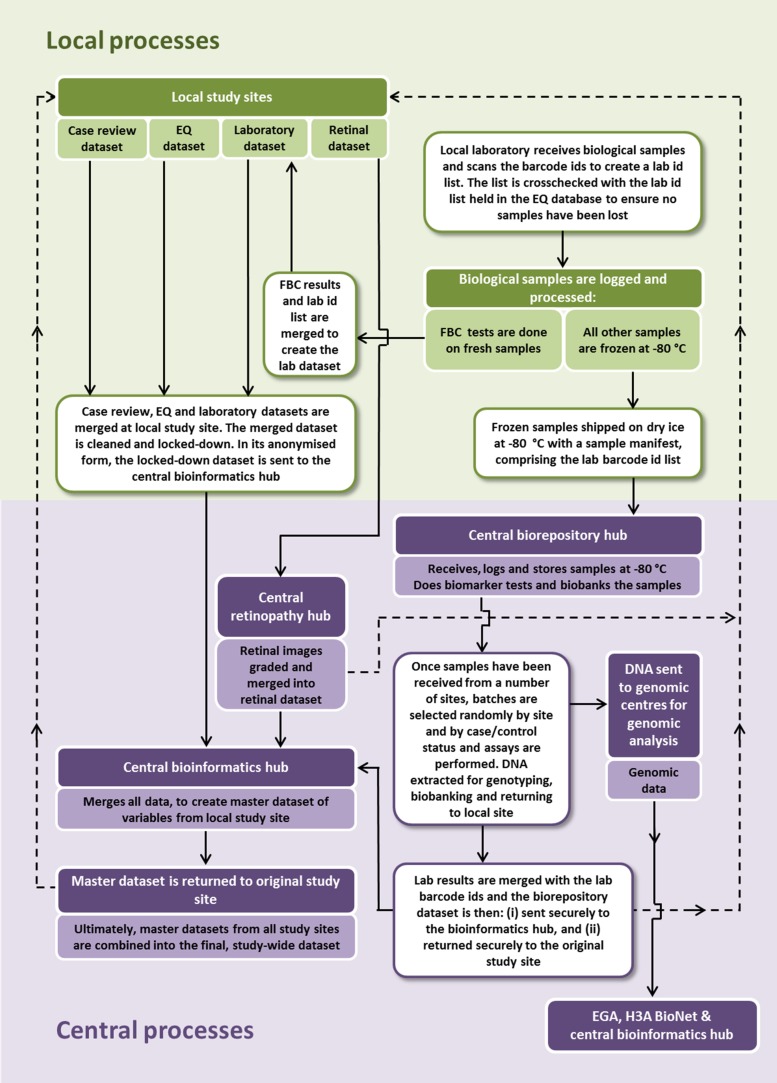
Overview of data management processes in the H3Africa type 2 diabetes study.

Data management for the study comprises processes for handling, storage and transfer of data at the local study sites, and at the various central hubs for this study (biorepository, retinopathy and bioinformatics). These processes are outlined in Fig. 5. Study sites will perform standardised quality control checks on their datasets before sending them to the central hub, for merging with laboratory results and retinal results to create a master dataset. Genomic data will be stored by the European Genomephenome Archive at the European Bioinformatics Institute, Hinxton, UK and in data centres in Africa – for example, the data centre of the APCDR in Uganda or the H3ABioNet in Cape Town. Curated and processed genomic data files will also be stored and managed at the central bioinformatics hub in South Africa. DNA may also be sent to, and stored, at other genome centres.

All these aspects of data management are described in more detail in standardised operating procedures which are available on request. There are also broader considerations and obligations for managing and sharing data across the wider H3Africa consortium, which are relevant to this study but fall outside the scope of this protocol paper.

### Statistical analysis plan

We will conduct site-specific analyses as well as study-wide pooled analyses. First, for each study site, means and medians with 95% confidence intervals (CIs) and interquartile range (IQR) will be calculated to summarise continuous risk factors. *T*-tests (or non-parametric tests where appropriate) will be used to compare results between cases and controls. Categorical risk factors will be summarised as frequencies and percentages with 95% CIs, and their prevalence in cases and controls compared using *χ*^2^ tests. Age- and sex-adjusted prevalence of T2D (and other diseases and risk factors) will be weighted appropriately to account for the multi-stage study design, and presented with 95% CIs [16, 17]. To determine risk factors and their relative contribution to the risk of developing T2D, odds of T2D will be modelled using logistic regression to obtain crude and adjusted ORs and 95% CIs. For outcomes that do not satisfy the rare disease assumption, a Poisson regression model will be used to model the probability of a given microvascular complication to obtain prevalence ratios (PRs). Population attributable fractions (PAFs) will be estimated to assess the relative contribution of each risk factor using the interactive risk attributable program software [18–20].

In addition to the above, we will pool site-level estimates of prevalence, ORs, PRs and PAFs to produce SSA-wide as well as regional (East, West and South SSA) estimates using random effects meta-analyses. A random effects model will be used because it accounts for between-site heterogeneity which we anticipate will be present in this study and will allow us to generalise the findings from this study to a wider population of similar studies [21, 22]. The type one error rate will be adjusted to account for multiple comparisons [23]. All statistical tests of hypotheses will be two-sided and significance tests performed at the 5% level.

### Disclosure of study results to participants

Study participants will be given their body mass index and blood pressure measurements in the field, at the point of data collection; survey participants will also receive their finger-prick blood glucose results. Where these results are abnormal, participants will be referred to their local clinic or hospital. Receipt of blood results is optional for participants, and individuals with abnormal venous blood results will be referred to their local clinic or hospital. Given the clinical implications of genetic profiles are unknown at the population and individual level, individual genetic data will not be disclosed to participants. This decision may be revisited in the future as the scientific community gains more insight into the practical clinical implications of genetic profiles of individuals. Aggregate genomic data will be disclosed to participants through meetings and seminars for study participants and local stakeholders in line with H3Africa public engagement policies and following site-specific procedures.

### Study governance

The study is led by the study principal investigator (PI – Professor A. A. Motala) and a scientific steering committee (SSC)^1^. The SSC comprises all local study site PIs and associated research partners. The study PI and SSC together are the custodians of the study, including samples and data, and are responsible for making critical decisions about the research direction of the programme. The study PI and SSC have complete autonomy with respect to dissemination, publication and scientific collaboration with the data and samples of the study. Advisory support and oversight will be provided by H3Africa governance structures.

### Ethical considerations

This protocol has undergone ethical review and received approval from institutional review boards and relevant national regulatory bodies at each of the participating study sites. Where required in order to obtain such approval, details of the protocol have been amended to satisfy local conditions, but without affecting the scientific integrity of the overall study. To further ensure that the study conduct is harmonised across the study sites, all aspects of the study – from generating the sampling frame, through to case identification, participant recruitment, data and sample collection and subsequent data management and sample processing – are detailed in standardised procedures; these are available on request.

Written informed consent is obtained from all study participants. The consent form covers the following activities: data collection by questionnaire and biophysical measures, sample collection and analysis including feedback of selected results, consent for DNA extraction and genomic analyses, consent for use of data and samples for future research, data deposition and sharing with H3Africa investigators and the wider scientific community via a process of managed access, and permission to re-approach participants for new studies.

## Discussion

This is a multi-site, multi-country population-based study integrating epidemiology and population genetics approaches to better understand the aetiology of T2D in SSA. A strength of this study is its broad geographical coverage and sample size. The study draws participants from West, East and Southern Africa, which enhances the relevance of its findings to populations across SSA. Secondly, with 6000 cases and 6000 controls, it will be the largest study of the epidemiology of T2D in SSA to date. This statistical resolution presents opportunity for uncovering new associations and risk factors. The study will include data on a broad range of exposures and phenotypes including chronic non-communicable and infectious diseases and risk factors, thus having the potential to address a wide-ranging set of research questions and the ability to provide biological insights into the variation in risk factors for a range of chronic diseases among adults in SSA. By aligning these data to genomic data, the study will be a platform for investigating genetic determinants of T2D and its complications. With a strong commitment to developing local research capacity, the study also provides opportunities for strengthening research infrastructure and analytical expertise for large-scale genomic and epidemiological studies across SSA [24].

A limitation of this study is its cross-sectional nature which means we will not be able to establish the temporal relationship between risk factors and outcomes. However, we plan to obtain consent from participants to re-approach them in the future, with the potential for establishing follow-up studies, prospective studies of disease incidence, and creating a platform for future research including studies of diabetes complications, interventional studies and clinical trials. Furthermore, the study has been designed specifically to allow for a broader use of population survey participants as control sets for many other disease case collections in the context of future studies. Banked biosamples are also available for future analyses. The study has no upper age limit for participants and is therefore well placed to investigate disease and risk factors in older populations. The study represents a diverse set of populations and environments. This design may lead to heterogeneity in the relationship between exposures and T2D risk. However, a key strength of this study is its use of agreed case definitions and standardised operating procedures across all study sites. With this level of harmonisation, the study will permit between-country and regional comparisons of disease burden and risk, and exploration of heterogeneity which may give deeper insight into contextual factors underlying disease aetiology.

Importantly, in a wider context, the study is well positioned for large-scale collaborative research – for example, it is part of the H3Africa consortium which has purposefully harmonised the assessment of phenotypes across its projects within this pan-Africa framework. In this way, the study represents a rich resource for providing evidence on the burden of T2D and other chronic diseases to inform the development of prevention and treatment strategies in the region. Further, the central aim of the study is to integrate epidemiological methods with population genomics and next-generation technologies. This approach has already provided new insights into the biology of chronic diseases and their risk factors in Western populations [9]. However, the relevance of many recent genomic findings to populations in SSA is not known. To date, genome-wide association studies in Africa have been limited to just a few thousand individuals across a limited number of cardiometabolic or infectious diseases and traits. Representing a major shift in the size of such studies, we aim to integrate genomic analyses into the proposed study, as well as to align such efforts with the wider H3Africa consortium. Given the marked genomic diversity among populations in SSA, understanding the genomic basis for chronic diseases and their risk factors in populations of African descent is likely to provide additional insights into the genetic determinants of chronic diseases and potential therapeutic strategies, including opportunities to discover novel disease susceptibility loci and variants and to refine (fine-map) association signals at new and existing disease and trait loci [25, 26]. The study is thus designed to maximise these opportunities for conducting large-scale genomic analyses and participating in genomics research globally.

### Collaboration and data sharing

In publishing this study protocol, the study investigators aim to maximise the scientific potential of this study, and welcome the fullest possible use of the data internationally. Data will be available to bona fide researchers and analysts who fulfil eligibility criteria, through a process of managed access. The study investigators also wish to encourage collaborations with other research institutes, stakeholders and policymakers. All data enquires should be made to the overall PI, Prof. Ayesha Motala (motala@ukzn.ac.za). Study documentation (protocol, participant information sheets and consent forms, questionnaires and standardised operating procedures) will be available via the APCDR website (data@apcdr.org).

## Supplementary material

For supplementary material accompanying this paper visit http://dx.doi.org/10.1017/gheg.2015.6. click here to view supplementary material
