# Supplementary material for: H3Africa multi-centre study of the prevalence and environmental and genetic determinants of type 2 diabetes in sub-Saharan Africa: study protocol
Source: Glob Health Epidemiol Genom. 2016 Mar 8;1:e5. doi: 10.1017/gheg.2015.6 (PMC5732581; doi:10.1017/gheg.2015.6)
Supplement: Supplementary file 1 [file S2054420015000068sup.zip › S2054420015000068sup003.docx]

**Title of project: A multi-centre study of the prevalence and environmental and genetic determinants of type 2 diabetes in sub-Saharan Africa**

**Prototype participant information sheet and consent form**

**IMPORTANT: For use by study participants with survey participants**

**IRB approval number (as required): ###**

**Date of Approval: ###**

**Name of Ethics Committee**: ………………………………………….……

1. **Introduction – why is this study being done?**

You are being invited to participate in this study of diabetes in Africans. Diabetes is a disease that occurs when there is too much sugar in the blood. The number of people in Africa with diabetes and other related conditions (such as high cholesterol, high blood pressure, and obesity) is increasing. We want to find out how common these diseases are, and why people develop them. We would like to understand more about the severity of these problems among African people, and if there are factors in the blood that make it more likely for someone to have these diseases or for them to run in their families.

Most people are unaware that they have these diseases. Evidence of these diseases may be found in the blood. We can also look for HIV and other infectious diseases in the blood. In this study, we want to find out more information about diabetes and other diseases in Africans. We will be asking around 12,000 people across Africa to take part in this study. This information may improve our understanding of health and disease in different populations in Africa. This could help us find new treatments for disease in the future.

This research study will last for five years and is being funded by the Wellcome Trust as part of the *Human Heredity and Health in Africa* (H3Africa) initiative.

1. **Names and affiliations of Principal Investigators**

The researcher in charge of this project is Prof. Ayesha Motala of the University of KwaZulu-Natal, Durban, South Africa. Dr. Clement Adebamowo of the Institute of Human Virology, Abuja, Nigeria is the researcher leading the study in Nigeria (insert relevant information for your setting here).

1. **What will happen to me if I decide to take part in the study?**

Your participation in this study should not take more than three hours of your time. If you choose to take part in the study, a trained interviewer will have a conversation with you about the study before enrolling you in the study. This conversation is called informed consent. You will be given this consent form to read. The interviewer will go over the consent form with you. You will have time to ask any questions you have about the study. You can request to take the consent form home and return with a decision whether to participate or not in the research.

After you give your consent to participate in the study, we will:

- Ask you questions about your health and lifestyle (for example, your diet, tobacco and alcohol consumption, physical activity, and medical history).
- Measure how much you weigh and how tall you are. We will also measure your waist and hip circumference and record your blood pressure.
- Take a fingerprick blood sample and blood samples (about four tablespoons of blood in total) from a vein in your arm.
- Collect a urine sample from you to test if diabetes affects your kidneys (albumin-creatinine ratio).

As part of the study we will ask you to fast overnight before we collect the blood samples. Fasting is when you intentionally refrain from eating or drinking anything other than water for a certain amount of time. For this study you must have not eaten anything for approximately ten hours. You may drink as much water as you wish during the fasting period. If the blood samples are taken after you have eaten something, they may provide unhelpful results because of the addition of outside material into your body from food or drink. Please remember, you will spend most of your fasting time asleep. You naturally fast when sleeping since you do not normally eat or drink. If you normally take medication in the morning, for the purposes of this study we ask that you refrain from taking your medication until after you have given blood. Please ensure that you bring your medication along with you to the study venue so you can take it after you have given blood.

When you give blood, the study team will begin by taking some blood from a vein in your arm, including a fasting glucose (sugar) test. You will then be asked to drink a special sugar drink. After 30 minutes, and again after two hours, the team will take a little more blood, to see how your body processes sugar in the blood. During the test you will be asked to sit quietly and not to eat but you will be allowed to drink water. This blood test may be able to tell if you are at risk of diabetes or already have it. We will give you a snack and drink once we have finished taking all the blood samples.

The tests on your blood will include those that look for diabetes, possible anaemia, high cholesterol and to see how well your kidneys and liver are working. We will also test for infectious diseases like HIV, hepatitis B, and hepatitis C. All information you provide and the results from the laboratory testing are kept confidential.

The blood tests will also examine your inherited (genetic) characteristics that you received from your parents. Information about inherited differences is found in the genetic material in your cells (we call this material DNA). We can use the DNA from your blood sample in order to look at the genetic material in your body. We will look for genetic differences that may have an effect on diabetes and other diseases. To better understand the origin of diabetes and other human diseases, factors in your blood that you inherited from your parents may also be used to study human history and how different people respond to different drugs.

1. **Storage of samples and access to data**

The samples that you donate to this study will be labelled with a unique code number so your name is not attached to the samples. Your samples will be sent for testing in the central laboratory for this study which is located in Africa, and for testing in laboratories located in other countries outside of Africa. It is necessary to send some of your samples to specialised laboratories in other countries such as the UK and US because some of the tests we want to do cannot be easily carried out in Africa. The information linking your name to the unique code number will be locked securely with strict access control and will not be made available to the researchers working on your sample. Remaining portions of your samples will be stored for an unlimited period of time for future use in other studies of health and disease.

To do more powerful research, it is helpful for researchers to share information they get from studying human samples. They do this by putting it into one or more scientific databases, where it is stored along with information from other studies. Researchers can then study the combined information to learn even more about health and disease.

If you agree to take part in this study, some of your genetic and health information might be placed into one or more scientific databases located in Africa and in other countries. Because this project is part of a larger research effort called H3Africa, your donated samples and health information will be stored in facilities maintained by appointed H3Africa investigators. Your name and other information that could directly identify you (such as address or social security number) will never be placed into a scientific database.

In addition, some of the samples and relevant information may be stored as part of a big collection or “biobank”. A biobank is a place that stores samples and other information so that researchers on this study and other scientists can use them in future unspecified research projects. We do not know all the different types of research or which scientists would do them, but appropriate measures (guidelines and rules) will be put in place to ensure that such studies comply with ethical standards.

1. **How will my information and samples be protected?**

All information which is collected about you in this study will be kept strictly confidential. To protect your privacy, we will use a unique code number to identify you and all information about you, including your blood and urine samples. Under no circumstances will your name, address or telephone number be given to any researchers. However, information on your ethnicity will appear alongside your genetic information. This is necessary to understand your genetic information better. We will keep your records and samples securely locked. For the current study, only research team members from this study will be able to look at the data and samples. Your name or any other facts that might point to you will not appear when we present this study or publish its results.

Your records and samples will then be securely archived for future research studies. Future research may be done by the current research team or by other research teams working in other countries. Only your records, samples and results which do not have your name or other identifiable information will be stored and shared with other research teams.

1. **Will I be informed about test results and findings from this research study?**

If you wish, you can choose to receive some of your test results (blood sugar level, for example). The results you can choose to collect are: …….[insert for each site]…… We will not provide the results of those tests related to your genes. This is because scientists do not yet fully understand the role of these genes in disease, and so the information may not be directly useful to you. If these circumstances change and there are genetic results that are meaningful and related to your health, these results will be provided to you after appropriate discussion about their meaning.

However, we intend to provide combined results of our research (results representing all participants, not identified individually) to participants through newsletters and other forms of communication.

1. **Will there be any benefits for me if I take part in this study?**

If you choose to collect your results and you find out that you have abnormal results, the study’s clinical feedback officer will recommend that you visit your local clinic where you may receive lifestyle advice or treatment to improve your health.

The information gained from this study will also help us to understand the causes of diabetes and other diseases among Africans, and could help us find new treatments for disease in the future. The information from this study may also help generate new knowledge about the history of your community and of your population, and may improve our understanding of health and disease in different populations in Africa.

1. **Will this study or the tests cause me any discomfort or put me at risk?**

You may experience a minor and temporary discomfort at the site of the blood draw. Possible risks might include bruising, pain, and infection at the puncture site. However these risks are very slight because the individuals who will draw your blood are experts and have been trained to cause as little discomfort as possible. We use only new sterile needles to collect blood.

You may find that some of the questions we ask make you feel uncomfortable. You are free to refuse to answer any questions. However, in order to have good results from the study, it is important that you attempt to answer all questions if possible. Our interviewers have been trained to treat each participant with respect and sensitivity. They will understand if you choose not to answer certain questions.

If you choose to receive some of your test results, knowledge of some of these results may cause some emotional stress or discomfort. However, our researchers will provide counseling and required referral to institutions/ hospitals where you can access information and the medical attention you need.

1. **Do I have to take part in this study?**

No. Participation in this study is entirely voluntary. If you initially consent to take part in the study but later decide you would like to withdraw from the study, you are free to do so. You do not need to give a reason, and this will not result in any penalties or loss of access to medical treatment or routine benefits that you are entitled to at this clinical facility.

If at any point, you would prefer to have your samples destroyed, you can let us know and we will destroy your samples. Please note that in such circumstances, certain data and samples that you previously provided might have been shared with other researchers, used in publications and studies or otherwise disseminated and these cannot be removed or altered.

1. **Will I be reimbursed for my participation?**

You will be reimbursed for your transportation and the time that you spent in participating in this research. You will be responsible for costs related specifically to your medical care. There is no other compensation available for your participation in this research.

1. **Additional information**

Sometimes, for many different reasons, a blood sample may not provide good information. We try to avoid this by using standardised techniques for collecting blood. However, if this does occur, we may get in touch with you and ask you to visit the clinic at your convenience so we can collect another blood sample for testing. You do not have to provide another blood sample at any time and this will not affect the care you receive in any way.

We would like to contact you in the future for possible new studies related to this study. If this happens, you will be provided with full information about these additional studies. You are under no obligation to take part in future studies and can choose not to be contacted.

You will be given a signed copy of this consent form for your records. If this research leads to commercial product, the rights to these products will be owned by the participating institutions. There is no plan to share such rights with research participants.

1. **Whom can I contact if I have questions or complaints about the study?**

We would like to answer all your questions. If you have any questions now, please ask us. If you have any questions in the future, you can contact:

Dr.……………..…….. from the University of/ Institution …….. (IRB Chairperson of the study site)

Email: …………………………………………….….

Phone: …………………………………………….….

Address: ………………………………………..……..

**Statement of person obtaining consent:**

“I confirm that I have fully explained this research, and provided complete and accurate information about it to……………………………………………………………….……. and he/she has confirmed that he/she fully understands the information, as well as risks and benefits associated with the study.”

Name:……………………………………………………….….. Signature:…………………….

Date/Time:…………….

**Statement of person giving informed consent:**

“I have read the information above, or it has been read to me. I have had the opportunity to ask questions about this study and all questions I have asked have been answered to my satisfaction. I consent voluntarily to participate in this study and understand that my consent means full participation in the study and access to my samples and data. I also understand that I have the right to withdraw at any time without it affecting my medical care in any way. I have received a copy of the consent form to be kept for my records.’’

Name: ……………………………………………………. Signature: …………………. Date/Time: …………..

Name of witness (where participant could not read the consent form and it had to be read

and interpreted) :

Name: ……………………………………………………. Signature: …………………. Date/Time: …………..
